# Whole-genome sequence and assembly of the sporogenic *Bacillus paralicheniformis* T7 strain with high proteolytic and amylolytic activities

**DOI:** 10.3389/fgene.2026.1720096

**Published:** 2026-01-21

**Authors:** Arman Mussakhmetov, Saniya Aktayeva, Arailym Sarsen, Asset Daniyarov, Bekbolat Khassenov, Ulykbek Kairov

**Affiliations:** 1 Laboratory for Genetics and Biochemistry of Microorganisms, National Center for Biotechnology, Astana, Kazakhstan; 2 Faculty of Natural Sciences, L. N. Gumilyev Eurasian National University, Astana, Kazakhstan; 3 Laboratory of Bioinformatics and Systems Biology, Center for Life Sciences, National Laboratory Astana, Nazarbayev University, Astana, Kazakhstan

**Keywords:** *Bacillus paralicheniformis*, Kazakhstan, oxford nanopore sequencing, proteolytic activity, whole genome

## 1 Introduction

The diverse enzymatic characteristics of *Bacillus* species render them advantageous for several applications, including food processing, agriculture, biomedicine, biofuel generation, hydrolysis, bioremediation, and natural polymer processing (Muras et al., 2021; Sharma et al., 2021; Zhao et al., 2021). Several bacillary strains exhibit elevated production capabilities (Contesini et al., 2018), with *Bacillus paralicheniformis* strains being prominent in the enzyme manufacturing sector. For instance, *B. paralicheniformis* MKU3 is recognized for its ability to produce proteases that effectively break down feather keratin (Santha Kalaikumari et al., 2019), while *B. paralicheniformis* BL. HK produces extracellular proteases used in the enzymatic processing of animal hides (Akhtar et al., 2024). Furthermore, *B. paralicheniformis* HR-1 and *Bacillus haynesii* HR-5, isolated from bottom sediments, are employed for the production of alkaline proteases applicable in the textile and leather industries (Thakor et al., 2025). Proteases obtained from *B. paralicheniformis* T7 also exhibit significant keratinase activity in the hydrolysis of avian feathers, wool, horns, hooves, and hides (Aktayeva and Khassenov, 2024b; Aktayeva and Khassenov, 2024a). Alongside proteases and keratinases, *B. paralicheniformis* strains produce α-amylases (Božić et al., 2020), phosphatases (Abdelgalil et al., 2021), esterases (Ganesh Kumar et al., 2021), and xylanases (Ngom et al., 2023). Specifically, the α-amylase produced by *B. paralicheniformis* ATCC 9945a effectively hydrolyzes starch without pretreatment (Božić et al., 2020). Additionally, the α-amylase from *B. paralicheniformis* GRA2 was integrated into a multienzyme preparation for the hydrolysis of food waste (Roslan et al., 2021). Meanwhile, *B. paralicheniformis* APSO efficiently produces thermostable alkaline phosphatase (Abdelgalil et al., 2021), and *B. paralicheniformis* strain G1, isolated from Arabian Sea sediments, secretes esterases that effectively biodegrade polystyrene (Ganesh Kumar et al., 2021).

In addition to enzymes, the antimicrobial peptides produced by *B. paralicheniformis* are of particular interest to researchers. For instance, bacitracin, produced by *B. paralicheniformis* UBBLi30, inhibits the growth of *Micrococcus luteus*, methicillin-resistant *Staphylococcus aureus* (MRSA), *Streptococcus pyogenes*, and *Propionibacterium acnes*, while additionally inhibiting biofilm formation by *M. luteus* and MRSA (Ahire et al., 2020). The antimicrobial peptide produced by *B. paralicheniformis* exhibits a bacteriostatic effect against *Salmonella typhi* and *Listeria monocytogenes* (Choyam et al., 2021) while remaining neutral against *Lactobacillus*. The advantage of these antimicrobial peptides is their resistance to degradation by physiological proteases or under high pH and temperature. Given these beneficial properties, such antimicrobial peptides may serve as viable alternatives to antibiotics as well as biopreservatives (Ahire et al., 2020).

Genomic methods are applied for the characterization of bacteria, enabling the identification of genes responsible for the biochemical and phenotypic features of a strain. Analysis of the *B. paralicheniformis* G1 genome revealed the presence of genes encoding serine, metallo-, and cysteine proteases. Most of the identified varieties were metalloproteases from the M20 and M50 families, along with serine proteases from the S8, S9, and S33 families (Santha Kalaikumari et al., 2021). Similarly, the *B. paralicheniformis* NBG-07 genome includes genes that code for various enzymes, including alpha-amylase, protease, cellulase, and laccase (Ramzan et al., 2024). Genomic sequencing of the polystyrene-degrading bacterium *B. paralicheniformis* G1 identified genes encoding monooxygenase, dioxygenase, peroxidase, esterase, and hydrolases capable of the degradation of synthetic polymers. Genes associated with bacterial motility and biofilm formation have also been identified in *Bacillus* species (Ganesh Kumar et al., 2021).

The present investigation focuses on the *B. paralicheniformis* T7 strain, isolated from Kazakhstan soil, which exhibits enhanced protease and keratinase activities. This strain also demonstrates α-amylase, esterase, phosphatase, and phytase activities, indicating that it may serve as a potential source for multienzyme production. Acquiring a comprehensive understanding of the strain’s biochemical features necessitates a genome-wide analysis. Such insights may then be applied in genetic engineering and in the formulation of strategies for improving the production of extracellular enzymes for biotechnological applications.

The objective of this work was to perform whole-genome sequencing and *de novo* genome assembly of the *B. paralicheniformis* T7 strain using Nanopore sequencing technology. A comprehensive investigation of the genome is expected to provide significant insights into the mechanisms of enzyme action. Moreover, this investigation will generate genetic data that is both valuable for future research and relevant for practical applications.

## 2 Materials and methods

### 2.1 Media, culture, and storage conditions

The *B. paralicheniformis* T7 strain was cultured on several media containing (*w*/*v*) lysogeny broth (0.5% yeast extract [Condalab, Madrid, Spain, Lot # 1702.00], 1% tryptone plus [Sigma-Aldrich, St. Louis, MO, United States, Lot # BCCB8073], 0.5% NaCl [Sigma-Aldrich, Lot # SZBE2960V0], pH 7.0), nutrient broth (Himedia, Mumbai, India, Lot # 0000044915), and feather medium (0.03% NaH_2_PO_4_, 0.035% Na_2_HPO_4_, and 0.75% feather powder, pH 7.0). The agar media used for the isolation and screening of the strain comprised (*w*/*v*) nutrient agar (TM Media, Rajastan, India, Lot # M3E1EV01), LB agar (lysogeny broth with 1.5% agar [TM Media, Lot # B1CA1HT01]), skimmed milk agar (2% skimmed milk, 0.1% NaCl, 1% tryptone plus, 1% agar), gelatin agar (0.4% peptone [TM Media, Product Code # 1506], 0.1% yeast extract, 1.5% gelatin, 1.5% agar), and feather agar (0.17% NaH_2_PO_4_, 0.035% feather powder, 1.5% agar). Sporulation was induced in Difco sporulation medium (*w*/*v*) (0.8% nutrient broth, 0.1% KCl, 0.012% MgSO_4_, 0.001% MnCl_2_, 0.005549% CaCl_2_, 0.15191 × 10^−3^% FeSO_4_, pH 7.2), Arret-Kirshbaum sporulation agar (0.6% pancreatic digest of gelatin, 0.4% casein enzyme, 0.3% g/L yeast extract, 0.15 g/L beef extract, 0.1% dextrose, 0.03% MnSO_4_, 1.5% agar, pH 7.0), and modified nutrient agar (0.28% nutrient agar, 0.01% CaCl_2_, 0.005% MnSO_4_, pH 6.9). The specific preparation methods are detailed in Aktayeva and Khassenov (2024a).

The strain was cultivated either in liquid broth at 30 °C–37 °C with shaking (150–200 rpm) for 10–16 h, or on solid agar medium at 30 °C–37 °C for 18–20 h. For short-term maintenance, the strain was stored on LB agar or nutrient agar plates at 4 °C for up to 30 days. For long-term storage, the cells were suspended in lysogeny broth supplemented with 50% sterile glycerol and stored at −80 °C. Storage at −80 °C for 5 years did not affect its characteristics, including growth conditions, sporulation, and enzymatic properties.

### 2.2 Isolation and identification of the strain

The strain was isolated from soil samples collected in Kazakhstan (42°54′00″N, 71°22′00″E) and was identified based on the morphological features of the colonies and cultures, as well as by light microscopic analysis (Bergey and Holt, 1994). For the identification of genetic features, the 16S rRNA gene was amplified using universal primers 27F and 1492R and subjected to Sanger sequencing (Sanger et al., 1977) using the BigDye Terminator v.3.1 Cycle Sequencing Kit (Thermo Fisher Scientific, Waltham, MA, United States) according to the manufacturer’s protocol. DNA fragments were separated using an ABI 3730xl automated sequencer (Applied Biosystems, Foster City, CA, United States). The obtained chromatograms were analyzed and compared with reference sequences using Vector NTI software v.11 (Thermo Fisher Scientific) and the NCBI database (http://blast.ncbi.nlm.nih.gov/Blast.cgi).

### 2.3 Enzyme assays

Keratinase activity was determined using azokeratin and keratin azure (St. Louis, MO, United States) as a substrate in 50 mM Tris-HCl (pH 9.0) at both 60 °C and 70 °C (Lin et al., 1992; Alane Beatriz Vermelho and Sabrina Martins Lage, 2013; Aktayeva and Khassenov, 2024b; Aktayeva and Khassenov, 2024a). Proteolytic activity was assessed according to Coêlho et al. (2016) using azocasein (Sigma-Aldrich) as a substrate in 50 mM Tris-HCl (pH 9.0) at 60 °С. Collagenase activity was evaluated according to Chavira et al. (1984) using azocoll (Sigma-Aldrich) as a substrate in 50 mM Tris-HCl (pH 9.0) supplemented with 1 mM CaCl_2_ at 37 °C. Milk clotting activity was measured following the method of Akishev et al. (2022) using cow milk as a substrate. Alpha-amylase activity was determined in 100 mM phosphate buffer, pH 6.0, at 85 °C via the reducing sugar method using potato starch as the substrate (Sigma-Aldrich) (Kiribayeva et al., 2024). Esterase activity was ascertained at 40 °C in 50 mM phosphate buffer, pH 7.0, according to Zhao et al. (2022) employing 4-nitrophenyl acetate (Thermo Scientific, Waltham, MA, United States) or 4-nitrophenyl octanoate (Thermo Fisher, Kandel, Germany) as a substrate. Alkaline phosphatase activity was assayed following the method of Abdelgalil et al. (2021) using p-nitrophenyl phosphate disodium salt hexahydrate (PanReacAppliChem, Darmstadt, Germany) as a substrate in 100 mM phosphate buffer, pH 10.3, at 70 °C. Phytase activity was assessed as described by Choi et al. (2001) using phytic acid sodium salt as a substrate in 100 mM Tris-HCl (pH 8.0) at 60 °C. All experiments were performed in triplicate. Enzymatic activity measurement data were derived from independent activity assays, with mean values, standard deviations (SD), and *p*-values calculated using GraphPad Prism version 8.0.1 (GraphPad Software, La Jolla, CA, United States, www.graphpad.com). All data are presented as means ± SD (*n* = 3).

### 2.4 Isolation and purification of endospores

Endospores were generated by culturing the strain on Arret-Kirshbaum sporulation agar or modified nutrient agar at 37 °C, and in Difco sporulation medium in a shaking incubator (150 rpm) at 37 °C. Cultivation time was determined by microbiological observation using Schaeffer-Fulton staining. Cultivation was stopped when the spore-to-cell ratio reached 1:1 or when spores predominated. Cells and spores were harvested by centrifugation. To remove the remaining vegetative cells, the suspension was heated for 20 min at 90 °C.

### 2.5 Antibiotic resistance

The strain’s antibiotic resistance profile was tested using the disk diffusion method as described by Matuschek et al. (2014). The following antibiotics were used: ampicillin (TM Media, 10 µg/disc, Lot # 041210), chloramphenicol (TM Media, 30 µg/disc, Lot # 0712109), ciprofloxacin (TM Media, 5 µg/disc, Lot # 0812110), clindamycin (TM Media, 2 µg/disc, Lot # 0012109), erythromycin (TM Media, 15 µg/disc, Lot # 0412109), gentamicin (TM Media, 120 µg/disc, Lot # 0012012), kanamycin (TM Media, 30 µg/disc, Lot # 0612110), nalidixic acid (TM Media, 30 µg/disc, Lot # 0922108), penicillin-G (TM Media, 10 µg/disc, Lot # 0422108), rifampicin (TM Media, 5 µg/disc, Lot # 0712109), streptomycin (TM Media, 10 µg/disc, Lot # 0912109), tetracycline (TM Media, 30 µg/disc, Lot # 0012109), tobramycin (TM Media, 10 µg/disc, Lot # 1212109), and cefazolin (HiMedia, 30 µg/disc, Lot # 0000156850).

### 2.6 Zymography and proteomic analysis

The culture supernatant of the strain was used for zymographic and mass spectrometric analysis. Zymographic analysis was performed accordingly to Aktayeva et al. (2022) using casein, keratin, and gelatin as substrates. To determine the specific protease classes, the following inhibitors were employed: phenylmethylsulfonyl fluoride, EDTA, E64, and Pepstatin A. Mass spectrometric analysis of the secreted proteome was performed using a Maxis Impact II Instrument (Bruker, Germany). The extracellular enzymes were identified via the Mascot platform.

### 2.7 Whole-genome investigation

The strain was cultivated in 10 mL of nutrient broth. The cells were harvested by centrifugation at 6,000 *g* for 7 min at 4 °C. Genomic DNA was isolated with a Genomic Wizard Purification Kit (Promega, Madison, WI, United States) following the manufacturer’s protocol. The quantity and quality of the DNA were determined using a NanoDrop OneC spectrophotometer (Thermo Scientific, Waltham, MA, United States) and agarose gel electrophoresis. The concentration obtained was 1.79 ng/µL, which corresponds to a yield of 38 μg of genomic DNA. Whole-genome libraries were generated using the Oxford Nanopore Technologies (ONT) Ligation Sequencing Kit (SQK-LSK109), which entailed the ligation of sequencing adapters following the manufacturer’s protocol (https://nanoporetech.com/document/gDNA-sqk-lsk109). The generated libraries were quantified using a Qubit 2.0 fluorometer (Invitrogen) and sequenced on the MinION platform using a FLO-MIN106 (R9.4.1) flow cell. Raw data underwent base calling using Guppy v3.4.1, followed by the removal of low-quality reads to ensure high-quality data.

**FIGURE 1:**
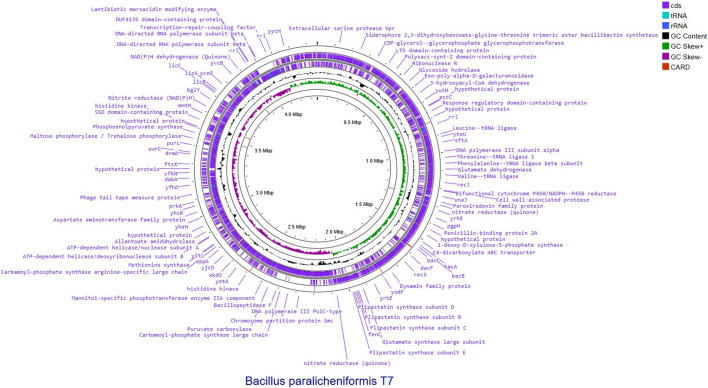
Circular genome representation of *de novo* assembled *Bacillus paralicheniformis* T7 genome. From the outside to the center: (1) predicted protein-coding sequences (CDSs) on the forward strand; (2) predicted protein-coding sequences (CDSs) on the reverse strand; (3) tRNA genes; (4) rRNA genes; (5) GC content plotted relative to the genome average; (6) GC skew, where positive values indicate G > C (green) and negative values indicate C > G (purple). Antimicrobial resistance genes identified using the CARD database are highlighted. The genome size and coordinate scale (in Mbp) are shown in the inner circle. The figure provides an overview of genome organization, gene distribution, and compositional features of the *B. paralicheniformis* T7 chromosome.

Sequencing generated a cumulative count of 149,880 sequencing reads with a median read length of 5,597 base pairs and an average read quality score of 11.98. The “*epi2me-labs/wf-bacterial-genomes*” module was employed for downstream analysis. *De novo* genome assembly was performed using the Flye v.2.9.1-b1780 algorithm (Kolmogorov et al., 2019). A single circular contig of 4,360,494 bp with a G + C content of 45.93% and a high mean contig coverage of 360X was generated (Supplementary File 1, Supplementary Table S1; Supplementary Table S2). Before annotation, the assembly was polished using Medaka (v.1.7.2) based on the same ONT reads. Genomic features were visualized via DNA Features Viewer. Additional genome annotation and circular genome visualization (Figure 1) were performed using Proksee (Grant et al., 2023). The PATRIC resource was accessed via the BV-BRC platform, which integrates and extends the former PATRIC database (Wattam et al., 2017; Olson et al., 2023). Genome completeness was assessed against the *bacillus*_odb12 database using BUSCO v.6.0.0 (Manni et al., 2021). The results showed that the assembly contained 97.2% complete BUSCOs (97.0% single-copy, 0.1% duplicated), 1.8% fragmented BUSCOs, and 1.0% missing BUSCOs (Supplementary File 1, Supplementary Table S3). The quality of the assembled *B. paralicheniformis* T7 strain genome was further verified using the CheckM2 tool (Supplementary File 1, Supplementary Table S4; Chklovski et al., 2023). Phylogenetic analysis was performed using CSI Phylogeny v.1.4 (Call SNPs and Infer Phylogeny) (Kaas et al., 2014) via the Center for Genomic Epidemiology (CGE) web service. As input, genome assemblies (FASTA format) of the studied strain and selected reference genomes were analyzed using a reference-based SNP-calling workflow implemented in CSI Phylogeny, with the *B. paralicheniformis* genome serving as the reference. Default alignment and SNP-calling settings were applied. SNPs were filtered based on a minimum depth at SNP positions of 10×, a minimum relative depth of 10%, a minimum distance between SNPs (pruning) of 10 bp, a minimum SNP quality score of 30, a minimum mapping quality of 25, and a minimum Z-score of 1.96. High-confidence SNPs were concatenated into a single alignment, and a maximum-likelihood phylogenetic tree was inferred using FastTree as implemented in CSI Phylogeny. Branch support values were estimated using the Shimodaira–Hasegawa-like (SH-like) approximate likelihood ratio test. The resulting phylogenetic tree was visualized and annotated using iTOL v.6 (Letunic and Bork, 2024).

### 2.8 Morphological and biochemical features of the strain

The present study provides the genetic sequence of strain T7, which was isolated from soil in Kazakhstan. The bacterial cells are Gram-positive, rod-shaped, motile, and capable of sporulation. Bacterial cells occur both singly and in chains. When cultured on LB agar at 37 °C for 16 h, the strain forms milky, irregular colonies (2–4 mm in diameter), characterized by a glossy surface and cloudy, opaque regions. When cultured in nutrient broth under aerobic conditions at 37 °C with shaking (150 rpm), the strain shows profuse growth within 24 h. During growth, the medium becomes cloudy and acquires a characteristic odor, and bacterial flakes form in the culture medium. A difficult-to-break film forms at the liquid-air interface. Based on these characteristics, the strain was classified as belonging to the genus *Bacillus*. Sequencing of the 16S rRNA gene identified the strain as *B. paralicheniformis* (100% identity). *B. paralicheniformis* T7 grew well in both nutrient and lysogeny broths. When cultivated on milk, keratin, and gelatin agar, clearance zones appeared, indicative of its protease, keratinase, and gelatinase activities. The strain grows across a wide temperature range of 30 °C–55 °C and a pH range of 6.0–8.5. These phenotypic and biochemical characteristics are typical of *Bacillus* species and are consistent with those of other *B. paralicheniformis* species (Dunlap et al., 2015). When cultivated in a bioreactor, *B. paralicheniformis* T7 achieves maximum proteolytic activity within 24 h, which is shorter than that reported for *Bacillus subtilis* KT004404 (Rehman et al., 2017) and *Bacillus* sp. CL33A (Ignatova et al., 1999). After 24 h of submerged fermentation in a bioreactor on minimal feather medium (0.3 g/L NaH_2_PO_4_, 0.35 g/L Na_2_HPO_4_, 7.5 g/L feather powder, pH 7.0), the culture supernatant demonstrated keratinase, protease, collagenase, milk-clotting, amylase, esterase, phosphatase, and phytase activities. The specific activities and their respective substrates are detailed in Table 1. For comparative context, the keratinase activity of *Bacillus licheniformis* ALW1 is 72.2 U/mL (Abdel-Fattah et al., 2018); the protease activity of *Bacillus cereus* FT and *Bacillus* sp. DPUA 1728 is 187 and 86.27 U/mL, respectively (Lima et al., 2015; Asha and Palanisamy, 2018); and the amylase activity of *B. licheniformis* 104 K is 163 U/mL (Kholikov et al., 2025). The high levels of keratinase, protease, and amylase, in combination with collagenase, amylase, esterase, phosphatase, and phytase activities, indicate that the *B. paralicheniformis* T7 strain has significant potential as a source of multienzyme preparations (Aktayeva and Khassenov, 2024a). This strain is capable of effectively hydrolyzing keratin-containing raw materials, particularly bird feathers, horns, hooves, wool, and cattle skin, resulting in the release of peptones and free amino acids (Aktayeva and Khassenov, 2024b). While keratinase and protease activity have also been observed in *B. paralicheniformis* MKU3, this strain differs from *B. paralicheniformis* T7 by the lack of collagenase activity (Santha Kalaikumari et al., 2019). During 5 days of cultivation on Difco sporulation medium, Arret-Kirschbaum agar, or modified nutrient agar, the strain produced endospores capable of withstanding a temperature of 121 °C and a pressure of 1.1 bar for 20 min. Additionally, this strain was sensitive to the following antibiotics: ampicillin, cefazolin, chloramphenicol, ciprofloxacin, clindamycin, erythromycin, gentamicin, kanamycin, nalidixic acid, penicillin, rifampicin, streptomycin, tetracycline, and tobramycin.

**TABLE 1:** Enzymatic activity of the supernatant from *Bacillus paralicheniformis* T7 after submerged fermentation. All measurements were performed three times independently, and the average of the three replicates was reported as the defined result with standard deviation (±SD).

Type of enzyme	Substrate	Activity (U/mL)
Keratinase	Keratin azure	63.6 ± 5.8
	Azokeratin	249.2 ± 7.9
Protease	Azocasein	715.7 ± 40.2
Collagenase	Azocol	23,6 ± 0,4
Milk clotting	Cow milk	5.8 ± 0.1
Amylase	Potato starch	176.1 ± 16.3
Esterase	p-nitrophenyl acetate	141.0 ± 0.01
	p-nitrophenyl octanoate	71.4 ± 0.02
Phosphatase	p-nitrophenyl phosphate disodium salt 6-hydrate	11.9 ± 0.6
Phytase	Phytic acid sodium salt hydrate	0.3 ± 0.03

Zymographic analysis using protease inhibitors—PMSF, EDTA, E64, and pepstatin A—complemented by proteomic studies, revealed that this strain secretes enzymes with molecular masses ranging from 20 to 60 kDa. These enzymes predominantly belong to the S8 and S41 families of serine peptidases (James, 1978) and the M14, M42, and M55 families of metallopeptidases (Hazra et al., 2012), with peak activity observed at 60 °C and pH 9.0. The enzyme extract of the strain has amylase activity, which reaches a maximum at pH 7.0 and at 85 °C. The strain’s high production capacity and bioreactor fermentation efficiency, along with its protease and keratinase activities, render it a promising candidate for industrial applications.

### 2.9 Genomic data

The whole genome of *B. paralicheniformis* T7 was sequenced to provide a comprehensive understanding of its genetic characteristics. Aseptic cultures of the strain were cultivated in 10 mL of nutritional broth in a shaking incubator at 37 °C and 150 rpm for 18 h. After cultivation, the cells were harvested by centrifugation at 6,000 *g* for 7 min at 4 °C.

**FIGURE 2:**
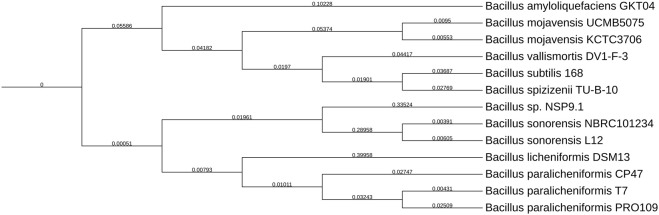
Phylogenetic tree depicting the evolutionary relationships among various *Bacillus* species. Branches are labeled with distance values indicating evolutionary divergence. Species include *Bacillus amyloliquefaciens, Bacillus mojavensis, Bacillus vallismortis, Bacillus subtilis, Bacillus spizizenii, Bacillus* sp. NSP9.1, *Bacillus sonorensis, Bacillus licheniformis*, and *Bacillus paralicheniformis*, each denoted with strain identifiers.

*De novo* assembly yielded a single circular contig of 4,360,494 bp exhibiting a high mean contig coverage of 360X (Supplementary File 1). Whole-genome assembly annotation via the PATRIC database identified a total of 4,652 protein-coding sequences, along with 82 tRNA molecules, 38 repetitive elements, and 24 rRNA molecules (Supplementary File 2). Among the identified proteins, 972 were classified as hypothetical, and 3,680 were assigned functional roles. Within the functional assignments, 1,099 proteins were associated with Enzyme Commission (EC) numbers, 926 with Gene Ontology (GO) terms, and 807 were mapped to KEGG pathways (Supplementary File 3). Comparative genomic analyses and phylogenetic tree reconstruction were performed using *Bacillus* genus reference strains retrieved from the NCBI database (Figure 2).

The whole-genome assembly for *B*. *paralicheniformis* T7 was deposited in NCBI GenBank under accession number CP124861 and BioProject number PRJNA967188. This assembly provides an extensive overview of the genetic information contained in this strain and facilitates the identification of the specific genetic components responsible for the observed morphological characteristics. The analytical pipeline encompassed bacterial culture, growth monitoring under various conditions, genomic DNA isolation, library creation, Oxford Nanopore sequencing, *de novo* genome assembly, genome annotation, and comparative genomic analysis. The final assembly is publicly available in NCBI GenBank to support additional comparative analyses and research.

## 3 Limitations

The genome sequence of the *B. paralicheniformis* T7 strain was generated using Oxford Nanopore technology, allowing for complete genome assembly. Nanopore sequencing enables the direct detection of nucleotides without the need for further DNA synthesis (Branton et al., 2008; Deamer et al., 2016) or imaging devices for nucleotide detection. By enhancing portability, this technology significantly reduces the initial cost of whole-genome sequencing (Branton et al., 2008). The fidelity of this sequencing method depends on the frequency at which a DNA strand is translocated through the pore (Mikheyev and Tin, 2014). This approach has exhibited robust performance in studies involving whole-genome sequencing (Mussakhmetov et al., 2024). Through the application of updated and validated bioinformatics pipelines and high sequencing coverage, the final genome assembly yielded a single circular contig.

## Data Availability

The data reported in the present paper are readily available and can be accessed from NCBI GenBank under the BioProject number PRJNA967188.
