# Supplementary material for: Whole-genome sequence and assembly of the sporogenic Bacillus paralicheniformis T7 strain with high proteolytic and amylolytic activities
Source: Front Genet. 2026 Jan 21;17:1720096. doi: 10.3389/fgene.2026.1720096 (PMC12867574; doi:10.3389/fgene.2026.1720096)
Supplement: Supplementary file 1 [file Supplementaryfile1.docx]

**Supplementary material S1**

**Table S1. Genome statistics of *Bacillus paralicheniformis T7 strain***

| **Genome Statistics** | |
| --- | --- |
| Contigs | [1](https://www.bv-brc.org/view/Genome/1386.2682#view_tab=sequences) |
| Genome Length | 4 360 494 |
| GC Content | 45.93 |
| Contig L50 | 1 |
| Contig N50 | 4 360 494 |
| **Genome Quality** | |
| Coarse Consistency (%) | 99.8 |
| Completeness (%) | 100 |

**Table S2. Read and assembly statistics of *Bacillus paralicheniformis T7 strain***

|  | Read count | Median read length (bp) | Mean read quality | Read data (Mb) | Largest contig (Mb) | Total length (Mb) | Reference length (Mb) | Mean contig coverage | # circular contigs |
| --- | --- | --- | --- | --- | --- | --- | --- | --- | --- |
| Bacillus paralicheniformis T7 | 149880 | 5597.0 | 11.98 | 1574.09 | 4.36 | 4.36 | 4.29 | 360.0 | 1 |

**Table S3. Genome assembly completeness of *Bacillus paralicheniformis T7 strain***

| Scores in BUSCO format | C:97.2%[S:97.0%,D:0.1%],F:1.8%,M:1.0%,n:778 |
| --- | --- |

**Table S4. Results of genome quality assessment using CheckM2 tool performed for *Bacillus paralicheniformis T7 strain* assembly**

| Name | Completeness | Contamination | Completeness Model Used | Translation Table Used | Coding Density | Contig N50 | Average Gene Length | Genome Size | GC Content | Total Coding Sequences | Total Contigs | Max Contig Length |
| --- | --- | --- | --- | --- | --- | --- | --- | --- | --- | --- | --- | --- |
| t7 medaka polished | 100.0 | 0.14 | Neural Network (Specific Model) | 11 | 0.873 | 4360494 | 292.923 | 4360494 | 0.46 | 4340 | 1 | 4360494 |
