# Supplementary material for: Whole-genome sequence and assembly of the sporogenic Bacillus paralicheniformis T7 strain with high proteolytic and amylolytic activities
Source: Front Genet. 2026 Jan 21;17:1720096. doi: 10.3389/fgene.2026.1720096 (PMC12867574; doi:10.3389/fgene.2026.1720096)
Supplement: Supplementary file 2 [file Supplementaryfile3.docx]

**Summary**

An assembled genome for Bacillus paralicheniformis T7 was submitted to the comprehensive genome analysis service at PATRIC^[^[^1^](file:///C:\Users\Ulykbek%20Kairov\Downloads\FullGenomeReport%20(1).html#citation-1)^]^. Based on the annotation statistics and a comparison to other genomes in PATRIC within this same species, this genome appears to be of Good quality. Details of the analysis, including genes of interest (Specialty Genes), a functional categorization (Subsystems) are provided below.

Genome Assembly

An assembled genome was submitted to the Comprehensive Genome Analysis service. This assembled genome had 1 contig, with the total length of 4,360,494 bp and an average G+C content of 45.93% (Table 1).


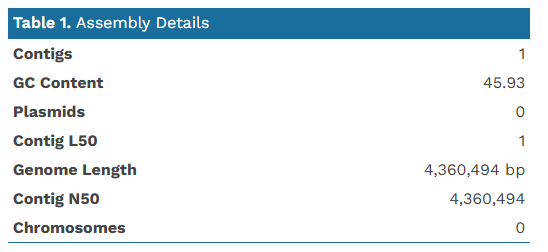


**Genome Annotation**

The Bacillus paralicheniformis T7 genome was annotated using RAST tool kit (RASTtk)^[^[^2^](file:///C:\Users\Ulykbek%20Kairov\Downloads\FullGenomeReport%20(1).html#citation-2)^]^ and assigned a unique genome identifier of 1648923.608. This genome is in the superkingdom Bacteria and was annotated using genetic code 11. The taxonomy of this genome is:

cellular organisms > Bacteria > Bacillati > Bacillota > Bacilli > Bacillales > Bacillaceae > Bacillus > Bacillus subtilis group > Bacillus paralicheniformis

This genome has 4,652 protein coding sequences (CDS), 82 transfer RNA (tRNA) genes, and 24 ribosomal RNA (rRNA) genes. The annotated features are summarized in Table 2.


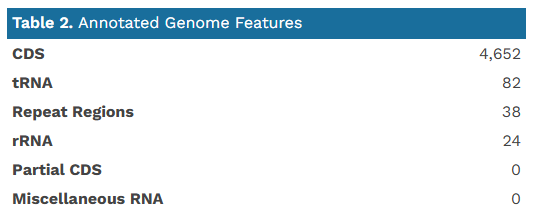


The annotation included 972 hypothetical proteins and 3,680 proteins with functional assignments (Table 3). The proteins with functional assignments included 1,099 proteins with Enzyme Commission (EC) numbers^[^[^3^](file:///C:\Users\Ulykbek%20Kairov\Downloads\FullGenomeReport%20(1).html#citation-3)^]^, 926 with Gene Ontology (GO) assignments^[^[^4^](file:///C:\Users\Ulykbek%20Kairov\Downloads\FullGenomeReport%20(1).html#citation-4)^]^, and 807 proteins that were mapped to KEGG pathways^[^[^5^](file:///C:\Users\Ulykbek%20Kairov\Downloads\FullGenomeReport%20(1).html#citation-5)^]^. PATRIC annotation includes two types of protein families^[^[^6^](file:///C:\Users\Ulykbek%20Kairov\Downloads\FullGenomeReport%20(1).html#citation-6)^]^, and this genome has 4,411 proteins that belong to the genus-specific protein families (PLFams) for , and 4,444 proteins that belong to the cross-genus protein families (PGFams).


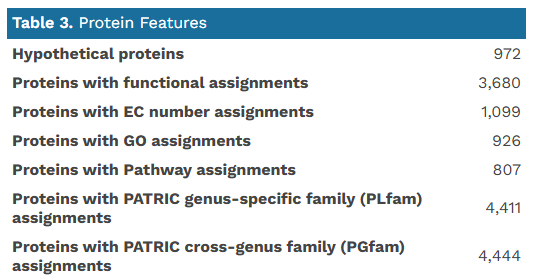


**Subsystem Analysis**

A subsystem is a set of proteins that together implement a specific biological process or structural complex^[^[^7^](file:///C:\Users\Ulykbek%20Kairov\Downloads\FullGenomeReport%20(1).html#citation-7)^]^ and PATRIC annotation includes an analysis of the subsystems unique to each genome. An overview of the subsystems for this genome is provided in Figure 2.


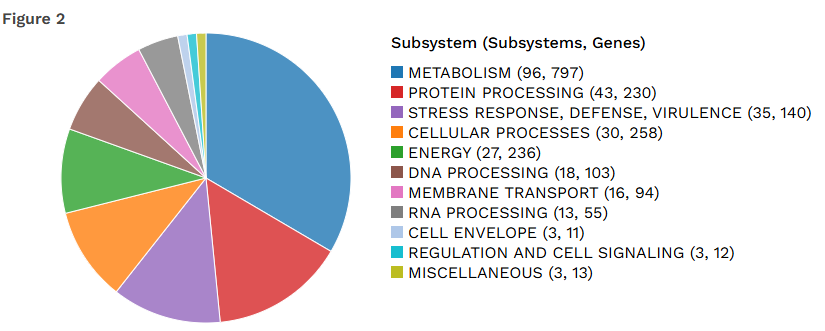


**Specialty Genes**

Many of the genes annotated in have homology to known transporters^[^[^8^](file:///C:\Users\Ulykbek%20Kairov\Downloads\FullGenomeReport%20(1).html#citation-8)^]^, virulence factors^[^[^9^](file:///C:\Users\Ulykbek%20Kairov\Downloads\FullGenomeReport%20(1).html#citation-9)^][^[^10^](file:///C:\Users\Ulykbek%20Kairov\Downloads\FullGenomeReport%20(1).html#citation-10)^]^, drug targets^[^[^11^](file:///C:\Users\Ulykbek%20Kairov\Downloads\FullGenomeReport%20(1).html#citation-11)^][^[^12^](file:///C:\Users\Ulykbek%20Kairov\Downloads\FullGenomeReport%20(1).html#citation-12)^]^, and antibiotic resistance genes^[^[^13^](file:///C:\Users\Ulykbek%20Kairov\Downloads\FullGenomeReport%20(1).html#citation-13)^]^. The number of genes and the specific source database where homology was found is provided (Table 4).


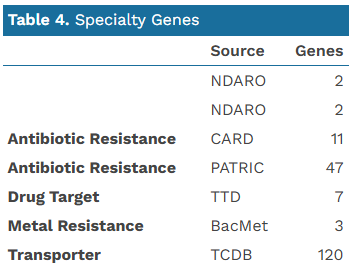


**Antimicrobial Resistance Genes**

The Genome Annotation Service in PATRIC uses k-mer-based AMR genes detection method, which utilizes PATRIC’s curated collection of representative AMR gene sequence variants^[^[^1^](file:///C:\Users\Ulykbek%20Kairov\Downloads\FullGenomeReport%20(1).html#citation-1)^]^ and assigns to each AMR gene functional annotation, broad mechanism of antibiotic resistance, drug class and, in some cases, specific antibiotic it confers resistance to. Please note, that the presence of AMR-related genes (even full length) in a given genome does not directly imply antibiotic resistant phenotype. It is important to consider specific AMR mechanisms and especially the absence/presence of SNP mutations conveying resistance. A summary of the AMR genes annotated in this genome and corresponding AMR mechanism is provided in Table 5.

**
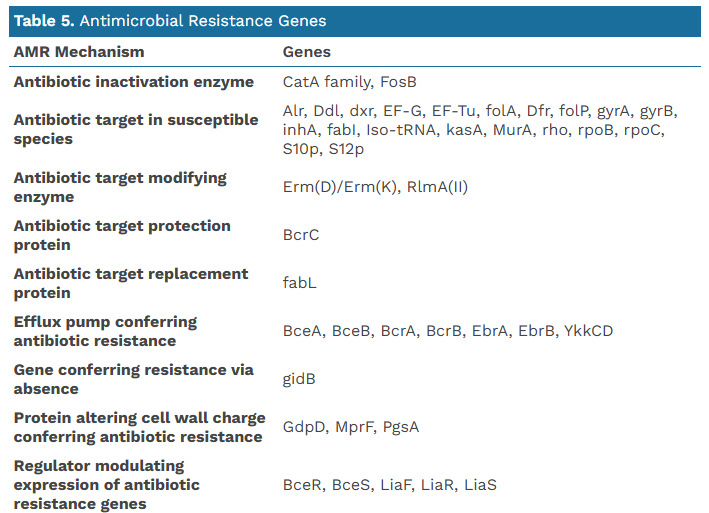
**

**References**

1. Wattam AR, Davis JJ, Assaf R, Boisvert S, Brettin T, Bun C, Conrad N, Dietrich EM, Disz T, Gabbard JL, et al. 2017. Improvements to PATRIC, the all-bacterial Bioinformatics Database and Analysis Resource Center. Nucleic Acids Res 45:D535-D542.
2. Brettin T, Davis JJ, Disz T, Edwards RA, Gerdes S, Olsen GJ, Olson R, Overbeek R, Parrello B, Pusch GD, et al. 2015. RASTtk: a modular and extensible implementation of the RAST algorithm for building custom annotation pipelines and annotating batches of genomes. Sci Rep 5:8365.
3. Schomburg I, Chang A, Ebeling C, Gremse M, Heldt C, Huhn G, Schomburg D. 2004. BRENDA, the enzyme database: updates and major new developments. Nucleic Acids Res 32:D431-D433.
4. Ashburner M, Ball CA, Blake JA, Botstein D, Butler H, Cherry JM, Davis AP, Dolinski K, Dwight SS, Eppig JT. 2000. Gene Ontology: tool for the unification of biology. Nature genetics 25:25.
5. Kanehisa M, Sato Y, Kawashima M, Furumichi M, Tanabe M. 2016. KEGG as a reference resource for gene and protein annotation. Nucleic Acids Res 44:D457-462.
6. Davis JJ, Gerdes S, Olsen GJ, Olson R, Pusch GD, Shukla M, Vonstein V, Wattam AR, Yoo H. 2016. PATtyFams: Protein Families for the Microbial Genomes in the PATRIC Database. Front Microbiol 7:118.
7. Overbeek R, Begley T, Butler RM, Choudhuri JV, Chuang H-Y, Cohoon M, de Crécy-Lagard V, Diaz N, Disz T, Edwards R. 2005. The subsystems approach to genome annotation and its use in the project to annotate 1000 genomes. Nucleic Acids Res 33:5691-5702.
8. Saier Jr MH, Reddy VS, Tsu BV, Ahmed MS, Li C, Moreno-Hagelsieb G. 2015. The transporter classification database (TCDB): recent advances. Nucleic Acids Res 44:D372-D379.
9. Mao C, Abraham D, Wattam AR, Wilson MJ, Shukla M, Yoo HS, Sobral BW. 2015. Curation, integration and visualization of bacterial virulence factors in PATRIC. Bioinformatics 31:252-258.
10. Chen L, Zheng D, Liu B, Yang J, Jin Q. 2016. VFDB 2016: hierarchical and refined dataset for big data analysis-10 years on. Nucleic Acids Res 44:D694-D697.
11. Zhu F, Han B, Kumar P, Liu X, Ma X, Wei X, Huang L, Guo Y, Han L, Zheng C. 2009. Update of TTD: therapeutic target database. Nucleic Acids Res 38:D787-D791.
12. Law V, Knox C, Djoumbou Y, Jewison T, Guo AC, Liu Y, Maciejewski A, Arndt D, Wilson M, Neveu V, et al. 2014. DrugBank 4.0: shedding new light on drug metabolism. Nucleic Acids Res 42:D1091-1097.
13. McArthur AG, Waglechner N, Nizam F, Yan A, Azad MA, Baylay AJ, Bhullar K, Canova MJ, De Pascale G, Ejim L. 2013. The comprehensive antibiotic resistance database. Antimicrobial agents and chemotherapy 57:3348-3357.
